# Prediction of severe CRS and determination of biomarkers in B cell-acute lymphoblastic leukemia treated with CAR-T cells

**DOI:** 10.3389/fimmu.2023.1273507

**Published:** 2023-10-03

**Authors:** Zhenyu Wei, Jiayu Xu, Chengkui Zhao, Min Zhang, Nan Xu, Liqing Kang, Xiaoyan Lou, Lei Yu, Weixing Feng

**Affiliations:** ^1^ Intelligent Systems Science and Engineering College, Harbin Engineering University, Harbin, China; ^2^ Shanghai Unicar-Therapy BioMedicine Technology Co., Ltd, Shanghai, China; ^3^ School of Chemical and Molecular Engineering, East China Normal University, Shanghai, China

**Keywords:** CAR-T cell therapy, cytokine release syndrome (CRS), biomarker, prediction, decision tree

## Abstract

**Introduction:**

CAR-T cell therapy is a novel approach in the treatment of hematological tumors. However, it is associated with life-threatening side effects, such as the severe cytokine release syndrome (sCRS). Therefore, predicting the occurrence and development of sCRS is of great significance for clinical CAR-T therapy. The study of existing clinical data by artificial intelligence may bring useful information.

**Methods:**

By analyzing the heat map of clinical factors and comparing them between severe and non-severe CRS, we can identify significant differences among these factors and understand their interrelationships. Ultimately, a decision tree approach was employed to predict the timing of severe CRS in both children and adults, considering variables such as the same day, the day before, and initial values.

**Results:**

We measured cytokines and clinical biomarkers in 202 patients who received CAR-T therapy. Peak levels of 25 clinical factors, including IFN-γ, IL6, IL10, ferritin, and D-dimer, were highly associated with severe CRS after CAR T cell infusion. Using the decision tree model, we were able to accurately predict which patients would develop severe CRS consisting of three clinical factors, classified as same-day, day-ahead, and initial value prediction. Changes in serum biomarkers, including C-reactive protein and ferritin, were associated with CRS, but did not alone predict the development of severe CRS.

**Conclusion:**

Our research will provide significant information for the timely prevention and treatment of sCRS, during CAR-T immunotherapy for tumors, which is essential to reduce the mortality rate of patients.

## Introduction

In recent years, with the continuous progress and application of cellular immunotherapy, chimeric antigen receptor (CD19)-modified T cells have shown great potential to treat hematological malignancies (1, 2). Children and adults with recurrent or refractory B-ALL treated by CD19 CAR-T cell therapy have achieved significant results, with a complete remission rate of 70% to 90% (3–5). However, patients often have serious side effects, involving the cytokine release syndrome (CRS). According to literature, 54% to 91% of patients may have different grades of CRS during treatment (6), according to CTCAE v5.0. If CRS is grade 3 or higher, it is considered a serious problem (4).

CRS is a high-risk factor associated with non-recurrent mortality (7). It is the main complication of CAR-T cell therapy, characterized by systemic inflammation, whose symptoms vary according to its severity, ranging from mild fever, fatigue, anorexia, nausea, vomiting, and headache to severe early high fever, hypotension, shock, and disseminated intravascular coagulation, leading to multiple organ dysfunction (8, 9). However, diagnosis and treatment of CRS may be delayed based on the available diagnostic criteria and severity grading system. Therefore, it is of clinical significance to understand CRS characteristics and related risk factors for effective management, which is mainly graded according to its severity and determined based on general symptoms, vital signs, and organ dysfunction. However, prognosis of CAR-T cell therapy is differentiated among individuals, thus specific biomarkers are needed to monitor and treat CRS (10). Biomarkers are defined as “characteristics that are objectively measured and evaluated as indicators of normal biological processes, pathogenic processes, or pharmacological responses to therapeutic interventions” (The Biomarker Definition Working Group of the National Institutes of Health, 1998).

CRS is a dynamic process of occurrence and development. CRS median time is two to three days after CAR-T cell infusion (range: 1 to 22 days) (11). Previous studies have identified several biomarkers that predict the development of adverse events after CAR-T cell therapy, which closely monitor patients at risk to receive timely preventive treatment (12, 13). However, limited classification standards accurately predict the occurrence of severe CRS.

To better predict CRS occurrence, we analyzed clinical data of 202 patients with B cell-acute lymphoblastic leukemia targeting CD19. We recorded cytokines, coagulation and biochemical indexes, blood routine, and a series of other biomarkers during continuous treatment of patients, which allowed us to develop novel studies and deepen people’s biological understanding of CRS that will further guide clinical practice.

In this manuscript, we will focus on the following aspects: 1) A comprehensive comparison of biomarkers between patients with and without sCRS to show critical details of its potential biology, 2) a significance analysis of the early contents of various biomarkers related to sCRS and a correlation analysis of their changes, 3) the sCRS prediction, classification model, and classification indexes with high sensitivity and specificity for adults and children, 4) the possible CRS grade of the next day from the clinical factor data of the patient the day before, providing high sensitivity and specificity classification indexes of adults and children, and 5) only from the initial value, we can determine the highest CRS level that the patient may reach, when returning for CAR-T treatment, and two decision tree models will be provided.

## Clinical description of patients

In this study, we included 202 patients with B-ALL, who consisted of 62 children from 0 to 25 years of age and 140 adults from 25 to 75 years of age. They were treated in the Affiliated Hospital of Suzhou Medical University in China. We evaluated the patient’s disease state before treatment and several lines of treatment. The first-line treatment refers to the first-round treatment after diagnosis. In this case, the selected treatment scheme was the one with the best clinical effect and the lowest side effects, whereas the second-line treatment is the one after the patient’s tumor progresses again. Compared to first-line treatment, second-line treatment demonstrated lower efficacy with 1 patient (0.5%) receiving no treatment, 25 patients (12.4%) receiving first-line treatment, 51 patients (25.2%) receiving second-line treatment, and 125 patients (61.9%) receiving third-line or higher treatments. For the disease state of patients, only 163 patients were recorded, among them the CR rate was 38% (62 individuals), and we also evaluated the curative effect. CR rate of 170 individuals recorded was 40% (68 patients). Of these patients, 95 (47%) did not relapse from B-ALL, 59 (28.7%) relapsed once, and 17 (8.4%) relapsed twice or more.

## CRS clinical description

Among 202 patients with B-ALL, 154 patients (76.2%) progressed to CRS, whereas most patients developed mild to moderate (grades 1 and 2; 109/202; 54%) or severe (grades 3 and 4; 45/202; 22.3%) CRS. For patients with fever, CRS onset was defined as the first onset of fever at 38.0°C after the infusion of CAR-T cells, and CRS termination was defined as no fever or use of vasoactive drugs within 24 hours. Among treated patients, 131 individuals developed fever symptoms and 23 patients progressed to CRS, without fever symptoms. Some patients showed serious organ toxicity, such as acute kidney injury, heart failure, moist rales at the bottom of the lung, among others, and many individuals developed CRS. **Table 1** contains some details of the study subjects. Some patients’ information is not recorded, so some information will contain fewer patients.

**Table 1:** Baseline Characteristics of the Patients.

Characteristics	Children (N=62)	Adult (N=140)	Total (N=202)
Sex			
**Female**	**22**	**76**	**98**
**Male**	**40**	**64**	**104**
Multiline treatment			
**median**	**3**	**3**	**3**
**range**	**1-9**	**0-13**	**0-13**
Number of recurrences			
**median**	**1**	**0**	**0**
**range**	**0-3**	**0-3**	**0-3**
**Transplant or not**	**13**	**30**	**43**
Extramedullary infiltration			
**yes**	**2**	**11**	**13**
**no**	**50**	**112**	**162**
Protoplast			
**median**	**3.25%**	**7.00%**	**5%**
**range**	**0-86%**	**0-94.5%**	**0-94.5%**
Dead or not			
**yes**	**9**	**22**	**31**
**no**	**53**	**94**	**147**

## CRS laboratory results

As shown in **Table 2**, baseline ferritin of most patients (N=136) increased (median 1102.5 mg/dL; range 32 to 11411 mg/dL). Regardless of the grade, the peak of ferritin in all patients was high but the median was significantly (P < 0.001) higher in patients with grades 3 and 4 CRS [grades 0 to 2 CRS (median 913 mg/dL; range 5.17 to 45,832) and 3 and 4 CRS (median 2100 mg/dL; range 38 to 281,253)]. Similar trends were observed in adults and children (**Table 2**). The ferritin peak in all patients with grades 3 and 4 CRS was higher than 10,000 mg/dL, which is considered to be sensitive and specific for macrophage activation/HLH syndrome in children (14, 15). In addition, baseline C-reactive protein (CRP) of most patients increased (median 7.4 mg/dL; range 1 to 425). Several patients were not evaluated for CRP at baseline. Similar to ferritin, most grades 3 and 4 CRS (median 59.4; range 1.2 to 960) and 0 to 2 CRS (median 8.535; the peak CRP of patients ranged from 0.818 to 288) were very high, and the CRS in grades 3 and was significantly (P < 0.001) different from that in grades 0 to 2. Similar trends were also observed in adults and children (**Table 2**). Although the peak levels of CRP and ferritin in patients with grades 3 and 4 CRS were higher than those in grades 0 to 2 CRS, CRP and ferritin did not improve CRS prediction in the first three days after CAR-T cell infusion (12).

**Table 2:** Clinical biomarkers associated with CRS (N =202).

			Total (N=202)		Children (N=62)		Adults (N=140)	
Biomarker		Total (N=202)	0-2 (N=157)	3-4 (N=45)	0-2 (N=47)	3-4 (N=15)	0-2 (N=110)	3-4 (N=30)
Blood coagulation items	**Plasma prothrombin time (PT)**	**12.6 (0.87-32.4)**	12.4 (0.9-32.4)^**^	13.15 (0.87-28.8)^**^	**12.8 (0.9-17.3)**	**13.2 (0.87-22.9)**	12.3 (0.96-32.4)^**^	13.0 (1.1-28.8)^**^
	**Activated partial thromboplastin time (APTT)**	**35.4 (4.07-378.2)**	34.45 (4.07-378.2)^**^	39.4 (20.9-86.1)^**^	35.3 (17.2-78.8)^**^	40.3 (26.0-79.8)^**^	34.0 (4.07-378.2)^**^	38.9 (20.9-86.1)^**^
	**Fibrinogen**	**3.35 (0.62-15.3)**	**3.31 (0.836-14.8)**	**3.52 (0.62-15.3)**	**3.163 (1.02-14.8)**	**3.61 (1.111-15.3)**	**3.4 (0.836-11.7)**	**3.51 (0.62-6.56)**
	**D-dimer**	**0.59 (0.012-99.3)**	0.47 (0.07-66.1)^**^	1.78 (0.012-99.3)^**^	0.395 (0.12-66.1)^**^	1.7 (0.27-50.0)^**^	0.51 (0.07-20.0)^**^	1.79 (0.012-99.3)^**^
Blood routine examination	**Red blood cell** **(RBC)**	**2.62 (0.14-336)**	2.7 (0.88-336.0)^**^	2.36 (0.14-204.0)^**^	2.985 (1.37-102.3)^**^	2.25 (1.29-4.49)^**^	2.64 (0.88-336.0)^**^	2.43 (0.14-204.0)^**^
	**Hemoglobin** **(HGB)**	**82 (0.65-158)**	85.0 (0.65-158.0)^**^	72.0 (36.0-131.0)^**^	90.0 (45.0-137.0)^**^	71.0 (38.0-128.0)^**^	83.0 (0.65-158.0)^**^	73.0 (36.0-131.0)^**^
	**White blood cell (WBC)**	**1.71 (0.01-397)**	2.85 (0.01-397.0)^**^	0.61 (0.01-69.79)^**^	1.78 (0.02-21.75)^**^	0.3 (0.01-6.47)^**^	**2.21 (0.01-397.0)**	**0.895 (0.01-69.79)**
	**Neutrophilic granulocyte percentage**	**0.731 (0.005-0.986)**	**0.739 (0.013-0.983)**	**0.667 (0.005-0.986)**	0.774 (0.035-0.983)^**^	0.537 (0.05-0.986)^**^	**0.7215 (0.013-0.98)**	**0.753 (0.005-0.984)**
	**Neutrophil count**	**1.22 (0.008-88.8)**	1.53 (0.01-88.8)^**^	0.415 (0.008-8.73)^**^	1.21 (0.01-19.99)^**^	0.28 (0.01-6.37)^**^	1.61 (0.01-88.8)^**^	0.515 (0.008-8.73)^**^
	**Percentage of** **lymphocytes**	**0.129 (0.002-0.991)**	0.121 (0.02-0.99)^**^	0.176 (0.004-0.991)^**^	0.1025 (0.005-0.892)^**^	0.423 (0.007-0.95)^**^	**0.135 (0.002-0.99)**	**0.1245 (0.004-0.991)**
	**lymphocyte count**	**0.17 (0.01-33.86)**	0.19 (0.01-33.86)^**^	0.11 (0.01-12.76)^**^	**0.15 (0.01-2.05)**	**0.15 (0.01-1.4)**	0.21 (0.01-33.86)^**^	0.1 (0.01-12.76)^**^
	**Platelet** **(PLT)**	**87 (0.006-527)**	100.0 (0.006-445.0)^**^	35.0 (2.0-527.0)^**^	107.0 (5.0-399.0)^**^	31.5 (4.0-175.0)^**^	97.0 (0.006-445.0)^**^	40.0 (2.0-527.0)^**^
	**Monocyte Percentage** **(MONO)**	**0.07 (0.002-0.952)**	0.072 (0.02-0.9)^*^	0.0575 (0.002-0.952)^*^	**0.067 (0.002-0.8)**	**0.066 (0.002-0.833)**	0.073 (0.003-0.9)^*^	0.05 (0.002-0.952)^*^
	**Monocyte count**	**0.11 (0.01-10)**	0.14 (0.01-6.79)^**^	0.04 (0.01-10.0)^**^	0.11 (0.01-3.53)^**^	0.03 (0.01-0.39)^**^	0.16 (0.01-6.79)^**^	0.04 (0.01-10.0)^**^
Biochemistry	**Sodium**	**139.7 (1.43-154.1)**	139.9 (2.33-151.6)^*^	139.2 (1.43-154.1)^*^	**140.0 (132.5-146.8)**	**139.4 (131.1-154.1)**	139.8 (2.33-151.6)^**^	139.0 (1.43-150.1)^**^
	**Potassium**	**3.81 (2.42-6.12)**	**3.82 (2.42-6.12)**	**3.74 (2.8-5.84)**	3.89 (2.66-6.12)^**^	3.635 (2.8-4.53)^**^	**3.79 (2.42-4.98)**	**3.76 (2.82-5.84)**
	**Chlorine**	**104.5 (1.94-119)**	**104.7 (1.94-119.0)**	**103.9 (85.0-117.1)**	**104.9 (92.5-112.2)**	**104.5 (94.7-115.4)**	**104.6 (1.94-119.0)**	**103.6 (85.0-117.1)**
	**Calcium**	**2.26 (0.93-147.3)**	2.28 (1.76-147.3)^**^	2.21 (0.93-2.54)^**^	2.3 (1.79-2.59)^**^	2.2 (1.57-2.54)^**^	2.26 (1.76-147.3)^**^	2.21 (0.93-2.52)^**^
	**Uric acid**	**210 (2.8-787.3)**	**214.2 (4.6-608.0)**	**196.45 (2.8-787.3)**	**208.0 (50.4-537.0)**	**208.9 (86.7-584.1)**	**215.0 (4.6-608.0)**	**195.0 (2.8-787.3)**
	**GLU**	**4.82 (1.78-323)**	4.71 (1.78-323.0)^**^	5.23 (1.98-15.96)^**^	4.555 (2.04-46.5)^**^	5.23 (3.91-8.14)^**^	4.8 (1.78-323.0)^**^	5.23 (1.98-15.96)^**^
	**Triglycerides**	**1.59 (0.39-96)**	1.555 (0.39-96.0)^*^	1.66 (0.45-15.15)^*^	**1.36 (0.39-46.1)**	**1.285 (0.45-9.09)**	1.65 (0.5-96.0)^**^	1.94 (0.72-15.15)^**^
	**Albumin**	**40 (1.4-426)**	40.7 (1.6-426.0)^**^	37.1 (1.4-51.9)^**^	42.5 (1.6-55.5)^**^	39.2 (25.2-51.9)^**^	40.0 (3.74-426.0)^**^	36.7 (1.4-47.8)^**^
	**ALT**	**19.6 (1.8-655.5)**	**19.5 (1.8-655.5)**	**20.0 (3.3-266.4)**	**19.9 (2.9-276.3)**	**17.6 (3.5-201.2)**	**19.4 (1.8-655.5)**	**21.2 (3.3-266.4)**
	**AST**	**19.5 (0.9-1599.3)**	19.0 (1.06-670.3)^*^	23.1 (0.9-1599.3)^*^	**18.4 (7.4-174.7)**	**16.9 (2.6-186.2)**	19.6 (1.06-670.3)^**^	26.0 (0.9-1599.3)^**^
	**ALP**	**80.45 (11.8-1420.4)**	79.6 (11.8-340.5)^**^	84.7 (26.2-1420.4)^**^	88.25 (26.0-242.4)^*^	71.5 (26.2-256.0)^*^	75.3 (11.8-340.5)^**^	102.6 (35.9-1420.4)^**^
	**γ-GT**	**53 (0-1131.3)**	43.95 (5.5-1051.9)^**^	108.0 (12.0-1131.3)^**^	27.95 (5.7-428.7)^**^	73.8 (12.3-725.7)^**^	53.05 (5.5-1051.9)^**^	133.0 (12.0-1131.3)^**^
	**LDH**	**193.7 (31-15930)**	186.6 (80.0-4531.9)^**^	219.5 (31.0-15930.0)^**^	**176.0 (97.8-2332.5)**	**181.0 (31.0-1075.2)**	192.75 (80.0-4531.9)^**^	243.0 (89.4-15930.0)^**^
	**Cr**	**51.1 (19-385)**	49.9 (19.0-137.23)^**^	59.25 (22.9-385.0)^**^	44.9 (19.0-126.4)^**^	64.0 (22.9-385.0)^**^	51.0 (26.0-137.23)^**^	58.0 (30.7-153.0)^**^
	**CRP**	**12 (0.818-960)**	8.536 (0.818-288.0)^**^	59.35 (1.184-960.0)^**^	6.635 (1.0-284.0)^**^	24.5 (1.78-409.0)^**^	9.86 (0.818-288.0)^**^	74.05 (1.184-960.0)^**^
	**Ferritin**	**1075.62** **(5.17-281253)**	**913.03** (5.17-45831.5)^**^	**2100.0** (37.58-281253.0)^**^	**646.875** (54.16-45831.5)^**^	**2383.29** (223.16-10574.04)^**^	**1051.33** (5.17-45000.0)^*^	**2001.0** (37.58-281253.0)^*^
	**Calcitonin original**	**0.1855 (0.02-100)**	0.157 (0.02-100)^**^	0.484 (0.026-23.52)^**^	0.1965 (0.021-3.68)^**^	0.396 (0.049-16.52)^**^	0.145 (0.02-100.0)^**^	0.527 (0.026-23.52)^**^
	**αHBDH**	**156.3 (66.9-14500)**	151.0 (67.0-2647.6)^**^	176.65 (66.9-14500.0) ^**^	**150.15 (78.7-1552.0)**	**143.0 (79.0-899.4)**	151.0 (67.0-2647.6)^**^	189.0 (66.9-14500.0)^**^
	**Prealbumin**	**221.5 (32.4-612.8)**	231.4 (32.4-499.3)^**^	176.25 (36.4-612.8)^**^	250.0 (77.7-499.3)^**^	185.9 (43.3-499.5)^**^	222.6 (32.4-488.0)^**^	174.8 (36.4-612.8)^**^
	**B type urine natriuretic peptide (BNP)**	**91.88 (5-35000)**	65.53 (5.0-7401.0)^**^	532.35 (5.1-35000.0)^**^	42.1 (5.0-2415.0)^**^	525.5 (5.1-35000.0)^**^	88.5 (5.0-7401.0)^**^	543.8 (5.7-25900.0)^**^
Cytokines	**IL-2**	**5.3 (0.3-273)**	4.6 (0.3-191.2)^**^	9.7 (0.8-273.0)^**^	5.0 (0.3-41.1)^*^	8.3 (1.1-185.6)^*^	4.4 (0.5-191.2)^**^	11.0 (0.8-273.0)^**^
	**IL-4**	**3.8 (0.3-156.9)**	**3.7 (0.3-31.0)**	**4.3 (0.5-156.9)**	**3.9 (0.5-24.0)**	**4.6 (0.7-156.9)**	**3.6 (0.3-31.0)**	**4.0 (0.5-53.2)**
	**IL-6**	**11.5 (0.8-14521.4)**	8.45 (0.8-4389.5)^**^	72.5 (1.2-14521.4)^**^	7.55 (0.8-1912.2)^**^	109.1 (2.0-7620.1)^**^	8.7 (1.0-4389.5)^**^	66.1 (1.2-14521.4)^**^
	**IL-10**	**5.9 (0.2-2439.1)**	4.9 (0.2-1664.3)^**^	13.2 (1.2-2439.1)^**^	5.0 (0.9-310.2)^**^	9.8 (1.2-181.0)^**^	4.8 (0.2-1664.3)^**^	17.6 (1.2-2439.1)^**^
	**TNF-α**	**4 (0.1-922)**	**4 (0.1-41.8)**	**4.25 (0.2-922.0)**	**3.8 (1.2-41.8)**	**3.85 (1.6-15.5)**	**4.1 (0.1-18.4)**	**4.45 (0.2-922.0)**
	**IFN-γ**	**8.7 (0.5-5338.2)**	6.9 (0.5-1443.7)^**^	21.8 (1.0-5338.2)^**^	6.2 (1.0-1443.7)^**^	31.8 (1.5-3774.2)^**^	7.1 (0.5-543.8)^**^	20.85 (1.0-5338.2)^**^
	**IL-17A**	**5.3 (0.8-84.7)**	**5.45 (0.8-84.7)**	**5.05 (1.0-78.2)**	**10.45 (0.8-32.3)**	**5.1 (2.2-78.2)**	**4.7 (1.0-84.7)**	**5.0 (1.0-54.8)**
**Dosage**		**5000000 (300000** **- 20000000)**	**5000000 (300000** **- 20000000)**	**5000000 (1000000-18000000)**	**5000000 (300000** **- 20000000)**	**5000000 (1000000 -10000000)**	**5000000 (300000 -20000000)**	**5000000 (5000000- 18000000)**

Unless otherwise noted, shows the median of peak observed values for the time period from the start of treatment to the onset of the highest grade of CRS (scope).

*P<0.01, **P<0.001, Kruskal-Wallis test.

Consistent with common inflammation and hypotension, some factors related to tissue injury include the significant increase of alanine aminotransferase (ALT), aspartate aminotransferase (AST), lactate dehydrogenase (LDH), and creatinine (Cr), and the levels of these factors are useful means to predict severe toxicity (16). For most patients with CRS, clinical factors of patients with grades 3 and 4 CRS are significantly higher than those with grades 0 to 2 CRS (**Table 2**). Although their peak values are related to the severity of CRS, none of them predict it during the first three days (17–19). For example, the increase of serum LDH concentration reflects the high tumor load of B-cell malignant tumor and may be related to the aggressive disease dynamics (20). In addition, there is evidence increasing LDH levels may be related to the microenvironment of immunosuppressive tumors, which inhibits CAR-T cells function, leading to tumor immune escape (21, 22). LDH is a clinical biomarker of the tumor lysis syndrome (TLS) (23), which is directly related to tumor load (24). This shows that LDH peak level is related to high-level CRS (12). In addition, the median LDH level in patients with grades 3 and 4 CRS correlated with ferritin levels (25). Other studies have shown that the levels of CRP, serum ferritin, and D-dimer are related to severe CRS (26). Common indexes of coagulation abnormalities, including prolongation of prothrombin time (PT), activation of partial thromboplastin time (APTT), increase of D-dimer and hypoproteinemia, have been reported in CRS with grade ≥ 3 (27, 28). For the dose of CAR-T cells transfused back, according to the statistical results, although there was no relatively strong significance. However, according to the minimum value, the minimum dose of severe CRS was higher than that of patients without severe CRS.

## Correlation analysis of clinical factors

**Figure 1:**
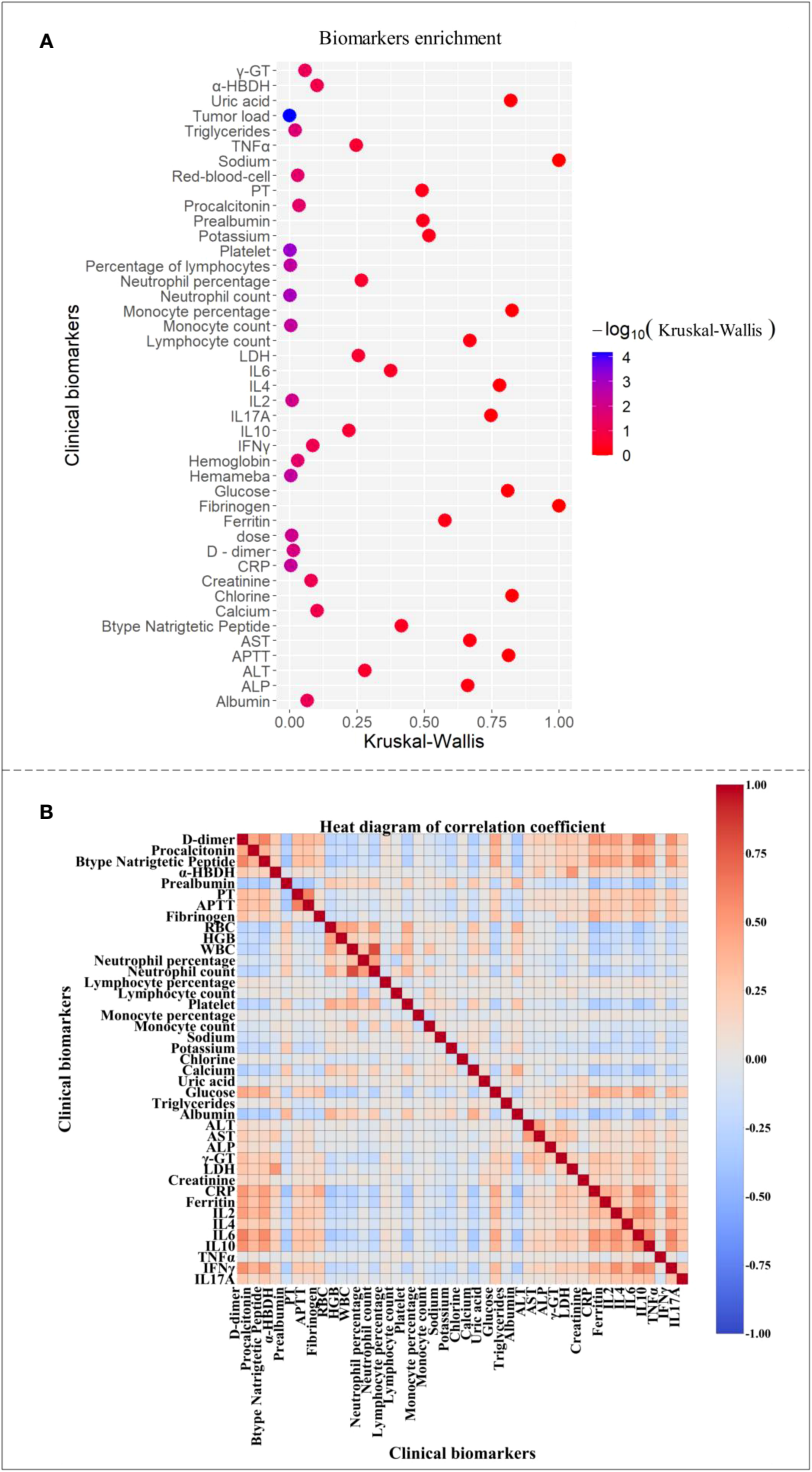
Clinical factor correlation map. **(A)** The clinical factors were significant. **(B)** Heat map of correlation coefficients between clinical factors.

We collected the data of clinical factors of each patient in the first day or two, sorted them from small to large, took the data of the first and last third, found the highest CRS grade of the patient, corresponding to these data in all time periods, and made significance analysis. The color related to significance in **Figure 1A** changed from blue to red, and it was found that the highest significance (P < 0.001) corresponded to tumor load and platelets count. Neutrophil, lymphocyte, leukocyte, and monocyte counts, CRP, dosage, and IL-2 were highly significant (P < 0.01), whereas D dimer, triglyceride (TAG), erythrocyte, hemoglobin, and procalcitonin were significant (P < 0.05). We also showed that the initial values of these 14 factors had a significant impact on whether patients have high-level CRS. Among them, platelets and hemoglobin decrease with the gradual increase of CRS grade, indicating that the lower the value of these factors, the more likely the patient will have high-grade CRS, whereas the higher the value of the other 12 factors one or two days ago, the more likely it will have high-grade CRS.

**Figure 1B** shows a thermogram of correlation coefficient between clinical factors of patients, where 1 indicates complete correlation, -1 indicates complete irrelevance, and the color from 1 to -1 gradually changes from warm to cool. We daily compared clinical factors of 202 patients and found the strong and weak correlation of some factors, among which the correlation between α -hydroxybutyrate dehydrogenase and lactate dehydrogenase was the strongest (0.99), indicating that the internal relationship between them was very close, and the changing trend was similar with the progress of CRS. Some studies evidenced that increased IL-6, CRP, and ferritin levels were related to the increase of prothrombin time and activated partial thromboplastin time to some extent (29). This is consistent with our analysis results. Moreover, in **Figure 1A**, the correlation coefficient between the factors with higher significance was also higher than that between the factors with lower significance, indicating that the biomarkers with greater influence on patients’ severe CRS grade are more closely related than those with other factors, which is beyond our expectation. Some of our conclusions agreed with those in most published articles, revealing the implicit relationship among patients’ clinical factors.

## Clinical factors spectrum

**Figure 2:**
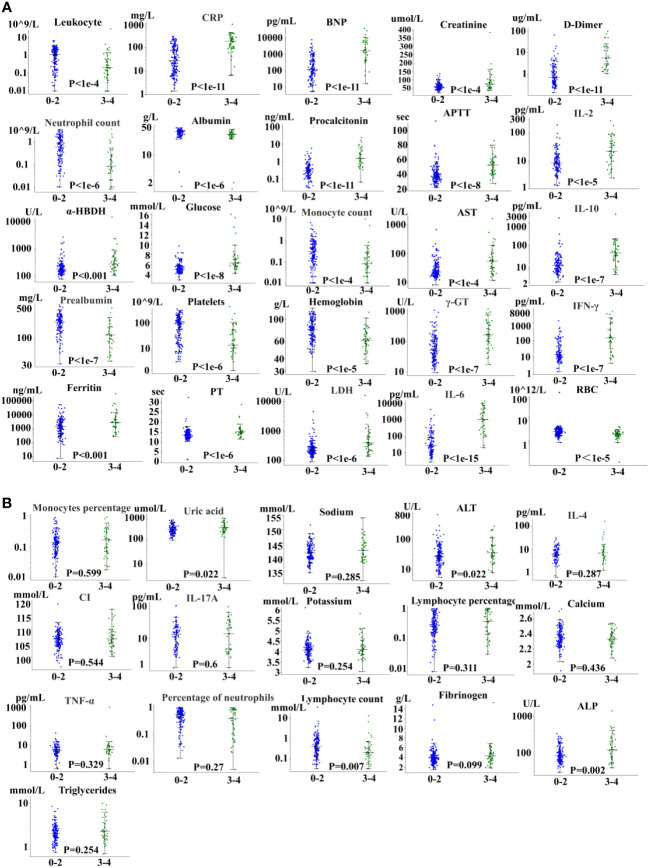
Severe CRS-related factors. **(A)** 25 factors with significant differences. **(B)** 16 factors with no significant difference.

Factors related to severe CRS are shown in **Figure 2**. We evaluated 41 related factors in 202 patients with B cell-acute lymphoblastic leukemia, including biochemistry, blood routine, cytokines, and coagulation factors. Biomarkers platelet, albumin, prealbumin, neutrophil count, neutrophil percentage, hemoglobin, potassium, red blood cells and white blood cells decreased with the increase of CRS grade, and we used the lowest value to represent the peak value. We can observe in **Figure 2** the peak difference of related factors during the period from the start of treatment to the occurrence of the highest-level CRS, including IL-2, IL-6, IL-10, and IFN-γ, and some clinical factors, such as D-dimer, CRP, procalcitonin, LDH, ferritin, among others. The peak levels of these factors were significantly (P < 0.05 by the Kruskal-Wallis test) different between 0-2 CRS and 3-4 CRS, and the P value obtained is significant. Moreover, we did not observe a significant difference in the severity of CRS among 16 related factors.

In addition, we found that the peak time of some cytokines and biomarkers in patients with severe CRS was earlier than that in patients without sCRS. Knowing the rising and falling time may not only improve the understanding of basic biology, but also have potential therapeutic significance. However, although IL-6 is the cytokine with the strongest correlation with sCRS, the early level of IL-6 (days 0 to 3) does not predict the occurrence of sCRS.

## Predictive modeling

**Figure 3:**
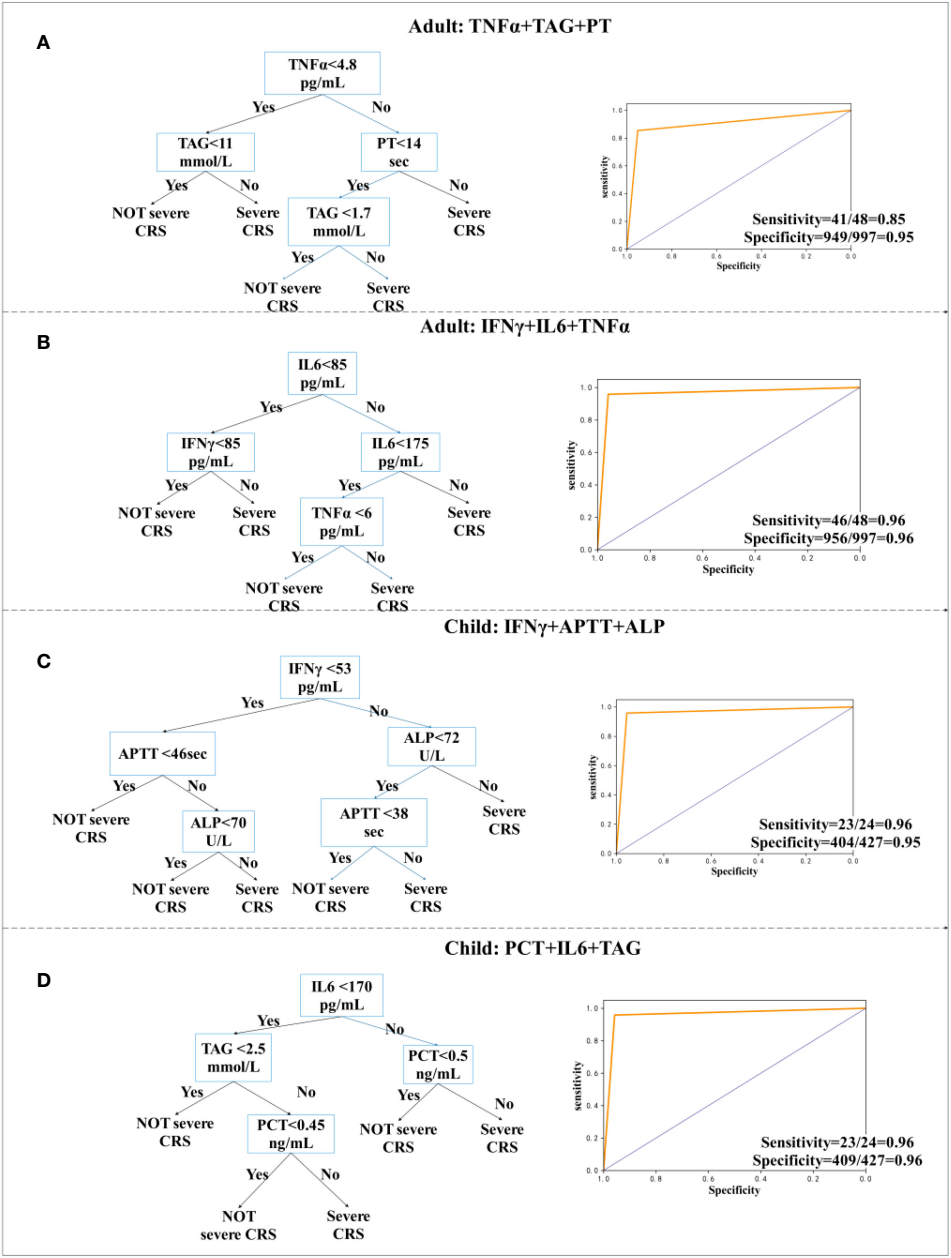
Decision tree prediction model for the day. **(A, B)** Decision tree prediction model for adults. **(C, D)** Decision tree prediction model for children.

**Figure 4:**
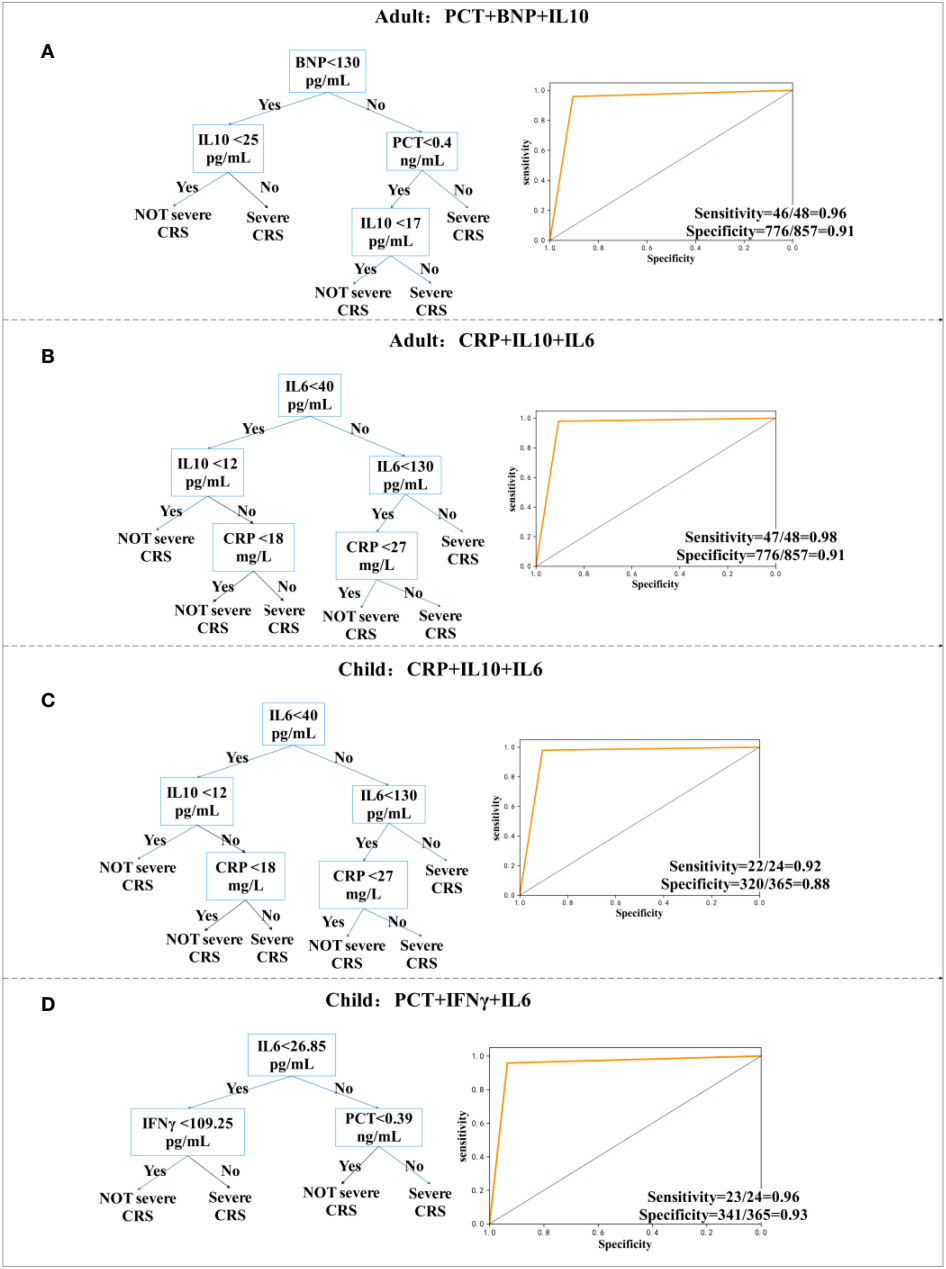
Decision tree prediction model one day in advance. **(A, B)** Decision tree prediction model for adults. **(C, D)** Decision tree prediction model for children.

**Figure 5:**
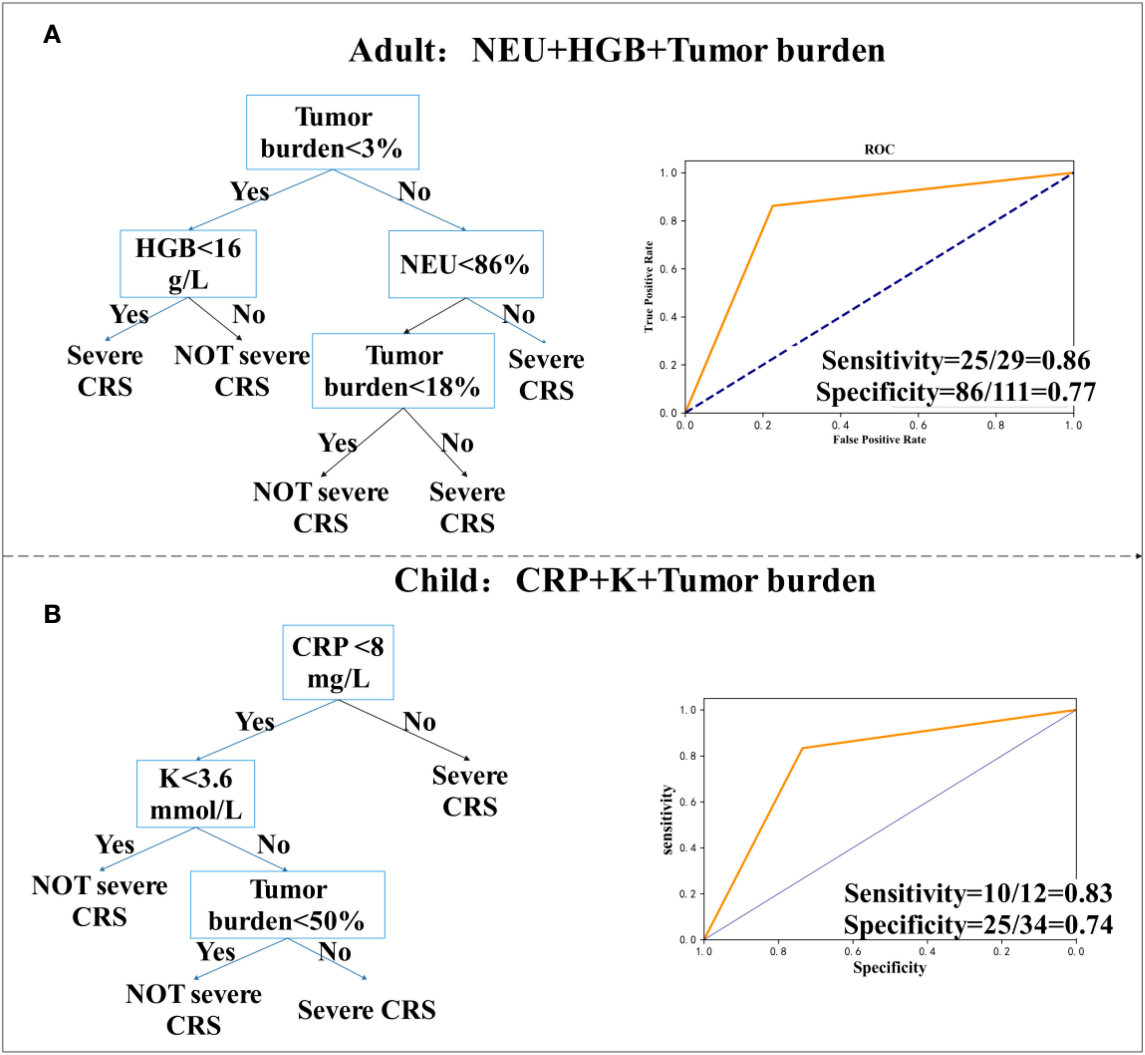
Decision tree prediction model of initial value. **(A)** Decision tree prediction model for adults. **(B)** Decision tree prediction model for children.

Based on the clinical data of 202 patients, we analyzed and provided classified prediction models, including five for adults and five for children. According to the data of current clinical factors, **Figure 3** lists the best decision tree models for children and adults, **Figure 4** provides the decision tree model for predicting one day in advance, and **Figure 5** is a model for predicting only the data of the previous day or two, when patients are transfused with CAR-T cells. We established the model by using the three-factor rule, and the model is accurate. According to the current data volume, the two-factor decision tree is not as good as the three-factor decision tree in terms of specificity and sensitivity, but the four-factor decision tree is easier to overfit, so the three-factor decision tree is more appropriate. These models are used to predict whether patients have sCRS and provide medical guidance in therapy.

For adults, TNFα, Triglyceride (TAG), and PT models were accurately predicted, with a sensitivity of 85% (95% CI, 0.94 to 0.96) (**Figure 3A**), whereas TNFα, IL-6, and IFN-γ models were accurately predicted, with a sensitivity of 96% (95% CI, 0.85 to 0.99) and specificity of 96% (95% CI, 0.94 to 0.97) (**Figure 3B**). Modeling for children was more accurate. The model using IFN-γ, APTT, and ALP has a sensitivity of 96% (95% CI, 0.77 to 1.0) and a specificity of 95% (95% CI, 0.92 to 0.96) (**Figure 3C**). We also observed a sensitivity of 96% (95% CI, 0.77 to 1.0) and a specificity of 96% (95% CI, 0.93 to 0.97), when using PCT, IL-6, and TAG (**Figure 3D**). According to the research, severity of CRS was related to IL-6 (30). In the cohort of children and adults, we provided two decision tree combinations related to IL-6.

We have explored the model of predicting one day in advance and provided two models for children and adults, which have the best sensitivity and specificity, and predict whether a patient will have sCRS the next day one day in advance, which facilitates a physician diagnosis one day earlier for timely intervention. For adults, we used a combination of PCT, B-type natriuretic peptide (BNP), and IL-10, whose sensitivity and specificity were 96% (95% CI, 0.85 to 0.99) and 91% (95% CI, 0.88 to 0.92), respectively. Furthermore, sensitivity and specificity were 98% (95% CI, 0.88 to 1.0) by using CRP, IL-10, and IL-6 models. For children, the model using CRP, IL-10, and IL-6 had a sensitivity of 92% (95% CI, 0.72 to 0.99) and a specificity of 88% (95% CI, 0.84 to 0.91), whereas the model using PCT, IFN-γ, and IL-6 had a sensitivity of 96%.

To determine the highest CRS grade that a patient may reach only from the initial value of the patient’s biomarker, we used the data of 202 patients, who were transfused with CAR-T cells for 0~1 day at the beginning. For children (N = 62), we found two models with good sensitivity and specificity. **Figure 5A** shows that in adults, the model for the combination of neutrophil percentage (NEU), MRD (which is considered as tumor load), and HGB, had a sensitivity of 86% (95% CI, 0.67 to 0.95), a specificity of 77% (95% CI, 0.68 to 0.85), and was high in CAR-T cells (31). According to previously published studies by Teachey and others, tumor load before infusion predicts sCRS (30, 32). However, tumor load may not be used to predict the model alone. In children’s cohort, we collected bone marrow from patients before transfusion to determine tumor load and used the decision tree model to evaluate if it has an important predictive variable in children’s cohort, but many experiments did not measure tumor load. For the combination of CRP, potassium (K), and tumor load, the sensitivity was 83% (95% CI, 0.51 to 0.97) and the specificity was 74% (95% CI, 0.55 to 0.86) (**Figure 5B**).

Using the patient’s initial value as predictor may be a tool for a physician to prevent the patient from having sCRS, which is life threatening.

## Biomarker analysis

The 10 models described above used TNFα, PCT, TAG, PT, IFN-γ, BNP, IL-6, IL-10, CRP, NEU, HGB, potassium, APTT, and ALP. We evaluated four cytokines, eight clinical factors, and a tumor load. Cytokines are often involved in inducing sCRS. If they are not under control, they may cause a cytokine storm in the later stage, which will damage organs and promote inflammatory responses. Organs damage will also produce a variety of biomarkers, thus the level of clinical factors is often a reaction to the severity of inflammation.

The cytokines IL-6, IL-10, IFN-γ, and TNF-α play a significant role in the genesis of CRS. A significant increase in endothelial activated cytokines (IL-6 and IFN-γ) and biomarkers (VWF and Ang-2) has been observed in patients with sCRS, which demonstrates that sCRS is characterized by endothelial cell activation (27). IL-10 is an anti-inflammatory cytokine, and IL-10 family cytokines play a key role in maintaining tissue homeostasis during infection and inflammation by limiting excessive inflammatory responses, up-regulating innate immunity, and promoting tissue repair (33).

Studies have shown that prealbumin, fibrinogen, and PCT are reactants in the acute phase of inflammation. The increase of blood fibrinogen content is considered an indicator of a pro-inflammatory state and a high-risk marker of vascular inflammatory diseases, whereas ICAM-1 signal affects the integrity of the endothelial cell layer and vascular permeability in a fibrinogen-dependent way (34). PCT level reflects the whole body inflammatory reaction and represents a prognostic biomarker for risk assessment of patients with severe infection and septicemia (35). However, prealbumin showed a significant change in serum concentration during inflammation, which negatively correlated with inflammation (36).

Serum and plasma BNP level in normal individuals is extremely low but its increase has a significant diagnostic value. It indicates whether a heart failure caused by inflammation has been corrected or is worsening. If treatment is effective, the level of BNP is significantly reduced. However, its increase usually indicates that patients´ heart failure is worsening (37). Some epidemiological studies have shown a correlation between the increased GGT activity level and sudden coronary heart disease (CHD) or CHD-related mortality (38). Furthermore, AST is common in liver function tests. It exists in many tissues of human body, particularly in myocardium, followed by liver, skeletal muscle, and kidney. In normal state, AST serum level is low. However, when cells of some organs are damaged, their membrane permeability and AST serum concentration increase (39). The content of D-dimer in patients’ plasma positively correlated with the severity of liver disease. The concentration of D-dimer is used as a marker to determine the degree of liver damage. Moreover, in sepsis, the level of D-dimer is used to evaluate the severity and prognosis of patients’ illness, and it has a certain effect on the evaluation of treatment effect (40).

## Discussion

In this study, we made new observations. Firstly, we comprehensively compared biomarkers between patients with and without sCRS, revealing important details of its potential biology. Secondly, we analyzed the early level increase of various biomarkers in patients with sCRS and the correlation between their changes. Thirdly, we developed a model to predict the development of sCRS, which enabled us for an early intervention to reduce the probability or mortality of CRS. Finally, we attempted to predict the CRS grade of patients one day in advance and only according to the initial value and provided an effective classification model. This is the first time that a model is used to predict at least one day in advance, which adds a new line of defense for the prevention and control of sCRS.

Our group’s data showed that, for example, the peak levels of LDH and ferritin have a strong correlation with sCRS. However, we did not find a strong relationship between the severity of CRS and its prognosis (30). It has been reported that the severity of CRS may be related to the tumor load during treatment (30, 32). Although this is in agreement with our study, our research evidenced that tumor load alone is not enough to predict which patients will develop sCRS, and tumor load should be classified and predicted together with other factors to have higher sensitivity and specificity. Despite some clinical factors are highly significant for patients with high-level CRS, it seems that this significance is not accurate enough to predict whether sCRS occurs.

We analyzed the influence of different patients’ early clinical factors on the later CRS grade. We observed that patients with sCRS had significant differences in the initial tumor load, platelets, neutrophil count, lymphocyte percentage, white blood cells, monocyte count, CRP, dosage, IL-2, D-dimer, TAG, red blood cells, hemoglobin, and procalcitonin, as compared with patients without sCRS. In terms of patient-specific factors, high tumor load, baseline thrombocytopenia, and elevated endothelial activation markers are related to the development of sCRS (41, 42). In patients with B-cell malignant tumor, receiving anti-CD19 CAR-T cells, a high tumor load in bone marrow has been identified as a risk factor for CRS (27), which is in agreement with our analysis. Regarding the cell dose of CAR-T reinfusion, providing patients with a lower cell dose may reduce the toxicity (4, 31), which is also consistent with our analysis.

Severe CRS is life-threatening. In our study, some patients died of CRS. Therefore, it is of great significance to predict and prevent the occurrence of sCRS. We showed three forms of prediction. Firstly, we predicted the patient´s CRS level, by analyzing his daily clinical data. Secondly, we predicted the CRS registration of the patient, one day in advance. Thirdly, we predicted whether the patient is prone to high-level CRS, according to his body´s biomarkers, 0 to 1 days after the patient is transfused with CAR-T cells. We also provided five classification models for children and five prediction models for adults. The prediction of the day provided physicians of an opportunity for an early diagnosis to determine a patient´s CRS grade of patients, allowing to stop the development of high-level CRS. For the prediction one day in advance, we found an overly sensitive and specific classification model for adults and children. The prediction one day in advance can enable doctors to prevent patients from reaching sCRS more accurately, ensure the life safety of patients, and minimize the effect of limiting treatment. For very early prediction, previous studies did not find any standard clinical biomarkers to help predict the severity of CRS, because many patients’ clinical biomarkers (such as IL-6, LDH, CRP, ferritin, among others) reached their peak after illness. When analyzing our own database, we found that the classification model with high sensitivity and specificity has the potential to determine whether the patient is prone to high-grade CRS, according to the initial cytokine levels in the early stage of treatment. However, early intervention and prevention of CRS will limit its efficacy to a certain extent, and the trial of early intervention will need to be carefully developed.

Cytokines are the most powerful laboratory biomarkers to predict CRS. However, in many clinical laboratories, it is impossible to rapidly evaluate cytokines. Therefore, our classification model allows other clinical factors to analyze and track the progress of CRS. After CAR-T cell therapy, CRS is not fully or accurately defined by different CTCAE scoring scales. Lee et al. and Davila et al. also published a CRS rating scale for patients treated with CAR-T cells (17, 32). Our CRS rating scale was compared with other published rating scales, and the grading system was remarkably similar. Therefore, the prediction model we developed was related to other grading systems. Regardless of the “score level”, our model identified patients who nearly presented life-threatening CRS complications.

Taken together, our study represents a comprehensive analysis of the clinical and biological manifestations of CRS, following CAR-T cell therapy. We have analyzed and identified clinical factors related to severe CRS and biomarkers that timely predict the development of severe CRS. The emergence of these models will enable patients with sCRS to be closely monitored and have the opportunity to start an active support treatment. At the same time, predicting the potential development of sCRS will prevent unnecessary cytokine intervention. Therefore, the model we produced, which was based on the use of biomarkers to predict sCRS, has direct clinical and therapeutic significance.

## Data availability statement

The original contributions presented in the study are included in the article/Supplementary Material. Further inquiries can be directed to the corresponding authors.

## Ethics statement

The studies involving humans were approved by The First Affiliated Hospital of Soochow University, Suzhou, China. The studies were conducted in accordance with the local legislation and institutional requirements. Written informed consent for participation in this study was provided by the participants’ legal guardians/next of kin. Written informed consent was obtained from the individual(s), and minor(s)’ legal guardian/next of kin, for the publication of any potentially identifiable images or data included in this article.

## Author contributions

ZW: Data curation, Formal Analysis, Methodology, Project administration, Supervision, Validation, Writing – original draft, Writing – review & editing. JX: Writing – review & editing. MZ: writing – review & editing. NX: writing – review & editing. LK: writing – review & editing. XL: writing – review & editing. LY: writing – review & editing. WF: Writing – review & editing. CZ: Writing – review & editing.
