# Corrigendum: Prediction of severe CRS and determination of biomarkers in B cell-acute lymphoblastic leukemia treated with CAR-T cells

**DOI:** 10.3389/fimmu.2024.1379325

**Published:** 2024-02-08

**Authors:** Zhenyu Wei, Jiayu Xu, Chengkui Zhao, Min Zhang, Nan Xu, Liqing Kang, Xiaoyan Lou, Lei Yu, Weixing Feng

**Affiliations:** ^1^ Intelligent Systems Science and Engineering College, Harbin Engineering University, Harbin, China; ^2^ Shanghai Unicar-Therapy BioMedicine Technology Co., Ltd, Shanghai, China; ^3^ School of Chemical and Molecular Engineering, East China Normal University, Shanghai, China

**Keywords:** CAR-T cell therapy, cytokine release syndrome (CRS), biomarker, prediction, decision tree

In the published article, there was an error in Supplementary and the names of the patients were erroneously included. The names have been removed from the supplementary materials to protect the anonymity and identity of the patients.

The supplementary material has been updated in the original article.

The authors apologize for this error and state that this does not change the scientific conclusions of the article in any way. The original article has been updated.

